# Disentangling primer interactions improves SARS-CoV-2 genome sequencing by multiplex tiling PCR

**DOI:** 10.1371/journal.pone.0239403

**Published:** 2020-09-18

**Authors:** Kentaro Itokawa, Tsuyoshi Sekizuka, Masanori Hashino, Rina Tanaka, Makoto Kuroda

**Affiliations:** Pathogen Genomics Center, National Institute of Infectious Diseases, Tokyo, Japan; University of Helsinki, FINLAND

## Abstract

Since December 2019, the coronavirus disease 2019 (COVID-19) caused by a novel coronavirus SARS-CoV-2 has rapidly spread to almost every nation in the world. Soon after the pandemic was recognized by epidemiologists, a group of biologists comprising the ARTIC Network, has devised a multiplexed polymerase chain reaction (PCR) protocol and primer set for targeted whole-genome amplification of SARS-CoV-2. The ARTIC primer set amplifies 98 amplicons, which are separated only in two PCRs, across a nearly entire viral genome. The original primer set and protocol showed a fairly small amplification bias when clinical samples with relatively high viral loads were used. However, as sample’s viral load become low, rapid decrease in abundances of several amplicons were seen. In this report, we will show that dimer formations between some primers are the major cause of coverage bias in the multiplex PCR. Based on this, we propose 12 alternative primers in total in the ARTIC primer set that were predicted to be involved in 14 primer interactions. The resulting primer set, version N1 (NIID-1), exhibits improved overall coverage compared to the ARTIC Network’s original (V1) and modified (V3) primer set.

## Introduction

The realtime surveillance of pathogen genome sequences during an outbreak enables monitoring of numerous epidemical factors such as pathogen adaptation and transmission chains in local to even global scale [1, 2]. Since it was first identified in Hubei, China in December 2019 [3], the novel coronavirus, SARS-CoV-2, responsible for the atypical respiratory illness COVID-19, has become a major concern for the medical community around the world. The relatively large genome size of corona viruses (approx. 30 kb) and varying levels of viral load in clinical specimens have made it challenging to reconstruct the entire viral genome in a simple and cost-effective manner. In January 2020, a group of biologists comprising the ARTIC Network (https://artic.network/), designed 196 primers (98 pairs) (https://github.com/artic-network/artic-ncov2019/tree/master/primer_schemes/nCoV-2019/V1) for targeted amplification of the SARS-CoV-2 genome by multiplexing PCR. These primers and method were based on a primer design tool Primal Scheme and a laboratory protocol PrimalSeq that had been previously developed for sequencing outbreaking RNA virus genomes directly from clinical samples using portable nanopore sequencer or other NGS platforms [4, 5]. The ARTIC primer set for SARS-CoV-2 (hereafter, ARTIC primer set V1) is designed to tile amplicons across nearly entire sequence of the published reference SARS-CoV-2 genome MN908947.3 [6]. The 98 primer pairs are divided into two separate subsets (Pools 1 and 2), such that no overlap between PCR fragments occurs in the same reaction.

**Fig 1:**
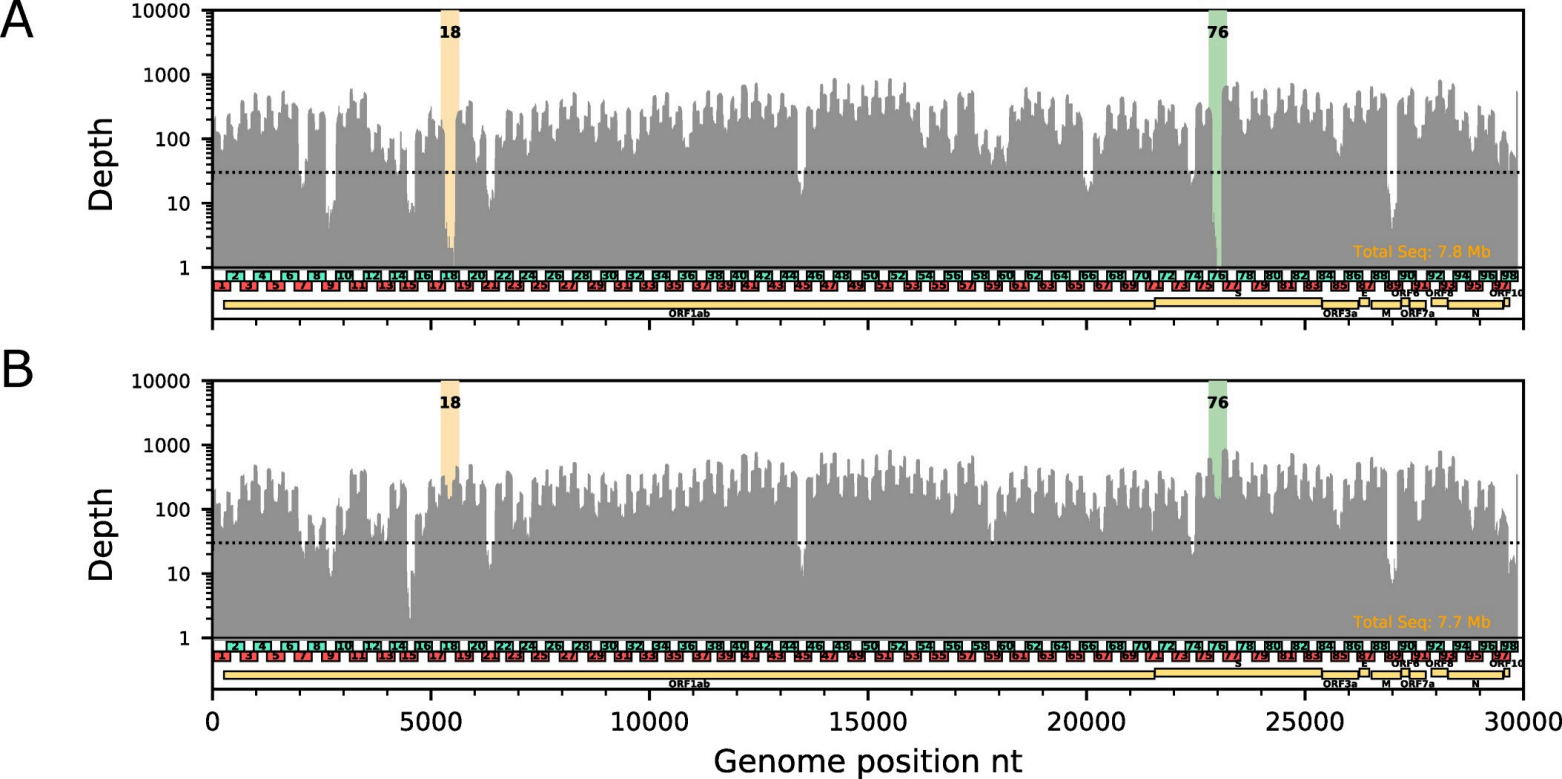
Dropout of the amplicon 18 and 76 due to primer interaction. Examples of depth plot for (A) original ARTIC primer set V1 and (B) V1 with 76_RIGHT replacement for the same clinical sample (previously deposited to GISAID with ID EPI_ISL_416596, Ct = 28.5, 1/25 input per reaction). Regions covered by amplicons with modified primer (76_RIGHT) and the interacting primer (18_LEFT) are highlighted by green and orange colors, respectively. For all data, reads were down-sampled to normalize average coverage to 250X. Horizontal dotted line indicates depth = 30. These two experiments were conducted with the same PCR master mix (except primers) and in the same PCR run in the same thermal cycler.

The ARTIC primer set V1 and the published protocol [7] worked well for samples with a relatively high viral load (Ct < 25 in clinical qPCR tests). For these samples, all designated amplicons are amplified with an acceptable level of coverage bias for subsequent NGS analysis. However, a gradual increase in the overall coverage bias was observed as a sample’s viral load decreased. Although this phenomenon is generally expected in such highly multiplexed PCR, the coverage for the two particular PCR amplicons, 18 and 76, decays far more rapidly than other targets (Fig 1A). In our experience, the low to zero depth for those two amplicons was the most frequent bottleneck for using the ARTIC primer set V1 to sequence all targeted genomic regions from samples with middle to low viral load (Ct > 27). Because the amplicon 18 overlaps the gene for nonstructural protein 3 (nsp3) in ORF1a, which is essential for assembly of the replicase–transcriptase complex, and the amplicon 18 overlaps the gene of spike (S) protein, which mediates receptor biding and virus-cell fusion [8], successful sequencing of those regions are potentially important for studies to understand evolution of the virus pathogenicity and infectivity. In situation with a high coverage bias in genome sequencing such as seen in Fig 1A, however, excessive sequencing efforts are required to obtain viral genome sequences with no or few gaps. Thus, minimizing the overall coverage bias will benefit the research community by both enabling more multiplexing in a given sequencing capacity and lowering the sequencing cost per sample.

In this report, we first show that the acute dropout of amplicons 18 and 76 was due to the formation of a single dimer between the forward primer for amplicon 18 and the reverse primer for amplicon 76. The replacement of one of the two interacting primers resolved the dropout of both amplicons. We further detected an additional 13 other potential primer interactions that may be responsible for low coverage in other regions covered by the affected amplicons. Our modified primer set, version N1 (NIID-1), which includes 12 primer replacements from the ARTIC primer set V1, yielded improved overall genome coverage in clinical samples compared to V1 and another modified primer set, V3. The results indicated that preventing primer dimer-formation is an effective measure to improve coverage bias in the ARTIC Network’s SARS-CoV-2 genome sequencing protocol, and may be applicable to other PrimalSeq methods in general.

## Results and discussion

In the original ARTIC primer set V1, PCR amplicons 18 and 76 were amplified by the primer pairs 18_LEFT & 18_RIGHT and 76_LEFT & 76_RIGHT, respectively. Those primers were included in the same multiplexed reaction, “Pool 2.” We noticed that two of those primers, 18_LEFT and 76_RIGHT, were perfectly complementary to one another by 10-nt at their 3′ ends (Fig 1). Indeed, we observed NGS reads derived from the predicted dimer in raw FASTQ data. From this observation, we reasoned that the acute dropouts of those amplicons were due to an interaction between 18_LEFT and 76_RIGHT, which could compete for amplification of the designated targets. Next, we replaced one of the two interacting primers, 76_RIGHT, in the Pool 2 reaction with a newly designed primer 76_RIGHTv2 (5′-TCTCTGCCAAATTGTTGGAAAGGCA-3′), which is located 48-nt downstream from 76_RIGHT. Fig 1A and 1B show the coverage obtained with the V1 set and the V1 set with 76_RIGHT replaced with 76_RIGHTv2 for cDNA isolated from a clinical sample obtained during the COVID-19 cruise ship outbreak, which was previously analyzed (EPI_ISL_416596) [9]. The replacement of the primer drastically improved the read depth in the regions covered by amplicons 18 and 76 without any notable adverse effects. The replacement of the primer 76_RIGHT improved coverage not only for amplicon 76, but also for 18 as well, supporting the hypothesis that the single primer interaction caused dropout of both amplicons.

**Fig 2:**
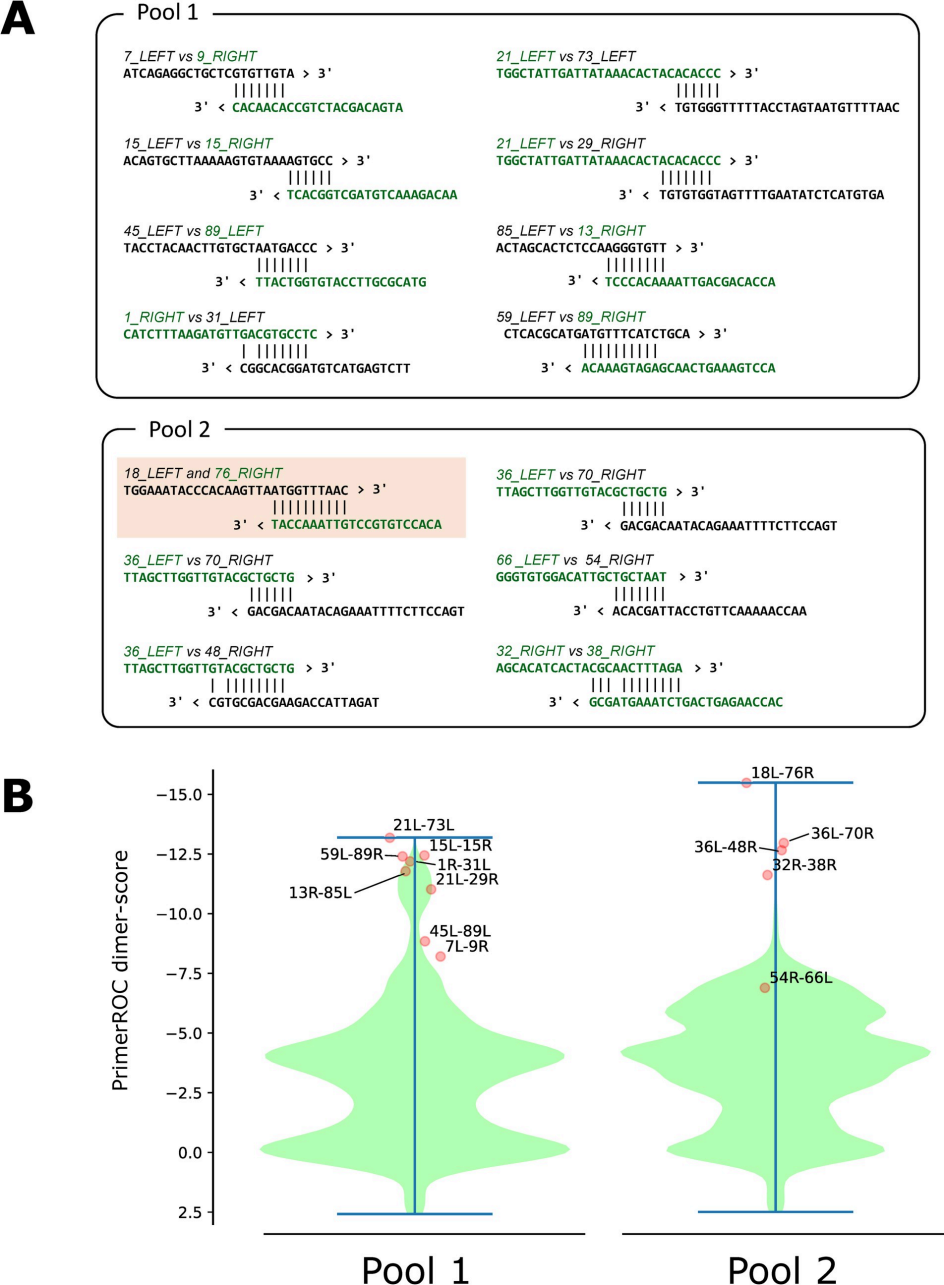
Predicted primer interactions. (A) The 14 predicted primer interactions subjected for modification in this study. Primers replaced in the N1 primer set (S1 Table) are shown in green. (B) Violin plots showing the distributions of dimer-scores (the nearest-neighbor ΔG adjusted by empirically determined penalty and bonus scores [10]) at all heterodimers in Pools1 and 2 reported by the PrimerROC algorithm (n = 4,743 for each pool). The scores of interactions depicted in Fig 2A are over plotted as scatter points.

Given this observation, we identified an additional 13 primer interactions using *in silico* analysis (Fig 2A and 2B). Those primer interactions predicted by PrimerROC algorithm [10], which gave the highest score for the interaction between 18_LEFT and 76_RIGHT among all 4,743 possible interactions, were likely involved in producing the low coverage frequently seen in our routine experiments. We, then, designed an additional 11 alternative primers, which resulted in a new primer set [ARTIC primer set ver. NIID-1 (N1)] including 12 primer replacements from the original V1 primer set (S1 Table). The N1 primer set eliminated all interactions shown in Fig 2A, and was expected to improve amplification of up to 22 amplicons (1, 7, 9, 13, 15, 18, 21, 29, 31, 32, 36, 38, 45, 48, 54, 59, 66, 70, 73, 76, 85, and 89). Alongside with this modification, the ARTIC Network itself released another modified version of primer set known as V3 in 24^th^ March 2020 [11] after we reported our result on the replacement of primer 76_RIGHT in a preprint [12]. The V3 primer set included 22 spike-in primers, which were directly added into the V1 primer set to aid amplification of 11 amplicons (7, 9, 14, 15, 18, 21, 44, 45, 46, 76, and 89).

**Fig 3:**
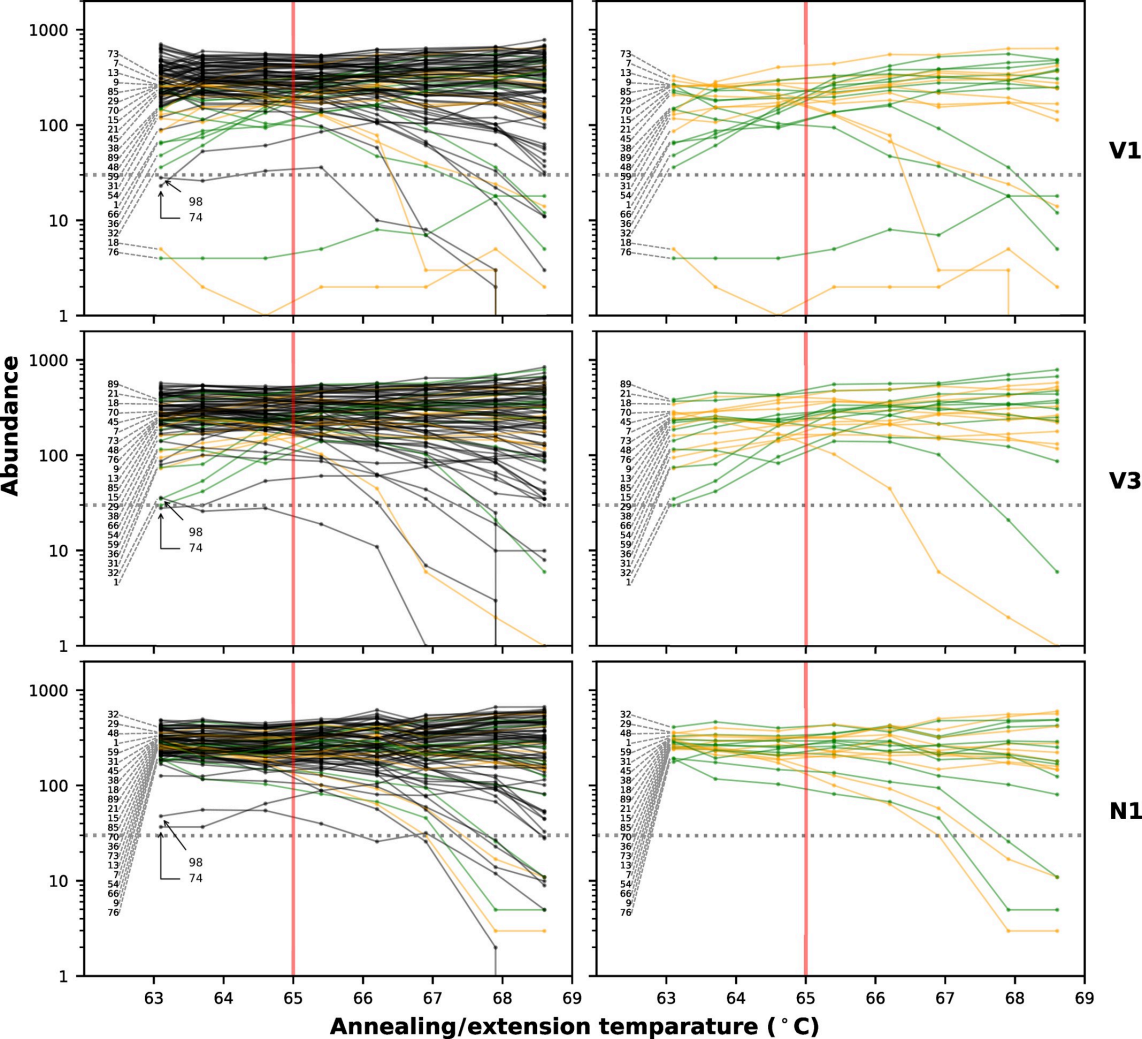
Responses to different annealing/extension temperatures. Abundance of 98 amplicons at 8 different annealing/extension temperatures with the three different primer sets on a same clinical sample (previously deposited to GISAID with ID EPI_ISL_416584, Ct = 16, 1/300 input per reaction). For all data, reads were down-sampled to normalize average coverage to 500X before analysis. The green lines and points indicate the abundances of amplicons whose primers in V1 primer set were subjected to modification in the N1 primer set. The orange lines and points indicate the abundances of amplicons whose primers were not modified but predicted to be eliminated the adverse primer interactions in the N1 primer set. Other amplicons which were not subjected to the modification are indicated by black lines and points. The plots in the left column shows results of all 98 amplicons while only amplicons targeted by modification are shown in the plots in the right column. Horizontal dotted line indicates fragment abundance = 30. Red vertical lines indicate normal annealing/extension temperature, 65°C. All those experiments were conducted with the same PCR master mix (except primers) and in the same PCR run in the same thermal cycler.

**Fig 4:**
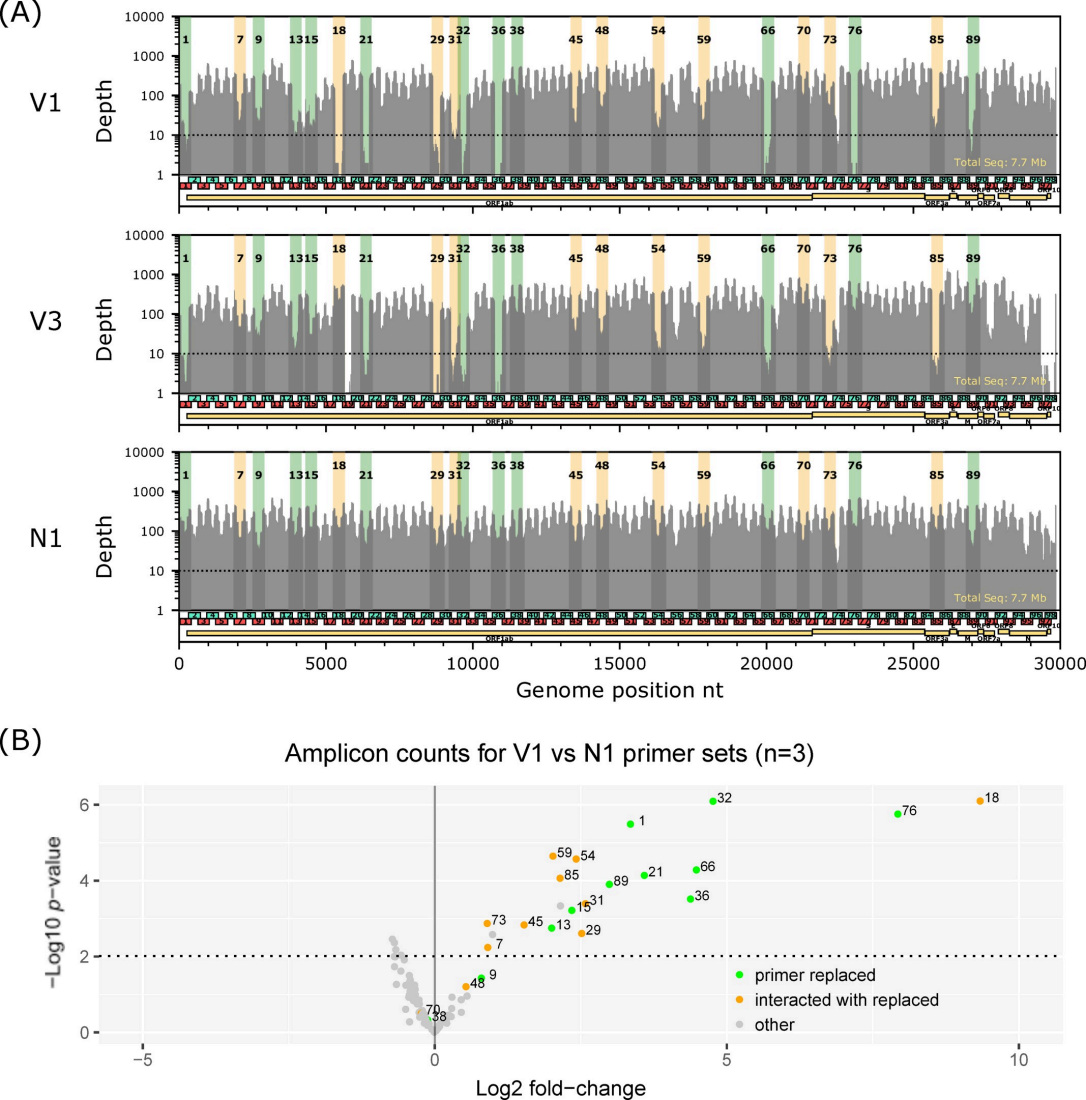
Performance comparison of the three multiplex PCR primer sets. (A) A depth plot of original (V1) and two modified ARTIC primer sets (V3 and N1) on a same clinical sample (previously deposited to GISAID with ID EPI_ISL_416596, Ct = 28.5, 1/25 input per reaction). Regions covered by amplicons with modified primers and with not modified but interacting primers are highlighted by green and orange colors, respectively. For all data, the reads were down-sampled to normalize average coverage to 250X. Horizontal dotted line indicates depth = 30. These two experiments were conducted with the same PCR master mix (except primers) and in the same PCR run in the same thermal cycler. (B) Volcano plot for coverages of all 98 amplicons in PCR using the primer set N1 compared with PCR using the primer set V1 among three clinical samples (shown in Fig 4A and S1 Fig). Points with green color indicate amplicons whose primer(s) was subject to replacement. Points with orange color indicate amplicons whose primers were not replaced but had been predicted to interact with either of the replaced primers as shown in Fig 2A.

We compared the performance of the original primer set (V1) and the two modified primer sets (V3 and N1) by observing their responses to different annealing/extension temperatures (*Ta*) in the thermal program (98°C for 30 sec followed by 30 cycles of 98°C for 15 sec and *Ta*°C for 5 min) using the gradient function of a thermal cycler. We surmised that this gradient temperature experiment would enable us to examine the dynamics of amplification efficiencies for each amplicon over varying annealing conditions. In general, amplicons suffering from primer interactions were expected to drop rapidly as *Ta* decreases. Fig 3 indicates the abundances of the 98 amplicons at eight different *Ta*, ranging from 63.1–68.6°C, using same dilution from a cDNA sample with high viral load (Ct = 16.0), which has previously been obtained from patients during the cruise ship outbreak sequenced (EPI_ISL_416584). With the V1 primer set, amplicons 18 and 76 exhibited extremely low coverage for all *Ta* values, with only a slight improvement above 67°C. In addition to those two amplicons, many other amplicons exhibited reduced coverage in these lower *Ta* range. Most of those amplicons were related to the predicted primer interactions depicted in Fig 2A. Although the dropout for amplicons 18 and 76 resolved with the V3 primer set, many amplicons still suffered low coverage in the low *Ta* region. Compared to the V1 and V3 primer sets, the modifications in the N1 primer set resulted in improved robustness of coverage over a broader *Ta* range for relevant amplicon. The improvement, however, made potentially weak amplicons 74 and 98 more apparent (Fig 3). The abundance of amplicons 74 gradually decreased with decreasing *Ta*, in contrast, the abundance of amplicon decreased with increasing *Ta*. These amplicons seemed equally weak in all three primer sets rather than specific in N1 primer set. So far, we have not yet identified interactions involving the primers for those amplicons. The gradient experiment also revealed relatively narrow range of optimal temperature for *Ta* for the V1 and V3 primer set, around 65°C, which was broaden for the N1 primer set. Nevertheless, while *Ta* = 65°C is a good starting point, a fine tuning of this value may help improving sequencing quality since an even slight difference between thermal cyclers, such as systematic and/or well-to-well accuracy differences and under- or overshooting, may affect the results of multiplex PCR [13]. Finally, we further compared the V1, V3 and N1 primer sets for three other clinical samples with relatively low viral loads using a standard temperature program (*Ta* = 65°C). In all three clinical samples (Fig 4A and S1 Fig), the N1 primer set showed the most even coverage distribution. The amplicons whose primer was prevented dimer formation (Fig 2A) by the primer modification in this study showed significantly improved coverage compared from V1 to N1 (Fig 4B).

The results of this study implicate importance of primer design which avoid dimer formation for PrimalSeq approach. We consider this insight will be helpful especially when revision of primer set (either for V3 or N1) become necessary in future to cope with the ever-increasing diversity of SARS-CoV-2 genomes (https://nextstrain.org/ncov).

## Conclusions

The formation of primer-dimers is a major cause of coverage bias in the ARTIC Network’s multiplex PCR protocol for SARS-CoV-2 genome sequencing. Eliminating these problematic primer interactions improves sequence coverage and will likely increase the quality of genome sequencing for SARS-CoV-2 and other viruses based on the PrimalSeq protocol.

## Materials and methods

### Design of alternative primers

The nCoV-2019_76_RIGHTv2 primer was redesigned using PRIMER3 software [14] set 65°C as optimal Tm and 20 nucleotides as optimal length. Other alternative primers were basically re-designed just by shifting their position several nucleotides toward the 5′ ends. For primer 36_LEFTv2 and 38_RIGHTv2, extension on the 5′ end were applied to set the medium dissociation temperature (*Tm*) predicted by the NEB website tool (https://tmcalculator.neb.com/) more than 63°C. Primer 66_LEFTv2 (Tm = 69°C) was designed by removing three nucleotides on the 3′ ends from 66_LEFT (Tm = 68°C) without position shift because this operation did not lower the Tm. See details of modifications on primers indicated in S1 Table. All new primers were assessed by PrimerROC [10] (http://www.primer-dimer.com/) to ensure no significant interactions with the remaining primers were predicted. All primer sequences included in the primer set N1 and information for their genomic positions were deposited to https://github.com/ItokawaK/Alt_nCov2019_primers. All primers used in this study were synthesized as OPC purification grade by Eurofins Genomics in Japan.

### cDNA samples and multiplex PCR

Four cDNA samples obtained from clinical specimens during the COVID-19 outbreak on a cruise ship February 2020 [9] were reused in this study. The use of human specimens was approved by the research ethics committee of the National Institute of Infectious Diseases (approval no. 1091). It was conducted according to the principles of the Declaration of Helsinki, in compliance with the Law Concerning the Prevention of Infections and Medical Care for Patients of Infections of Japan. The ethical committee waived the need for written consent regarding the research into the viral genome sequence. The personal data related to the clinical information were anonymized, and our procedure is not to request written consent for all patients suffering from COVID-19. The cDNA had been synthesized from RNA of SARS-CoV-2 positive pharyngeal swabs detected by the real time RT-PCR assay [15]. Reverse transcription was conducted using protocol published by the ARTIC Network [7], and then diluted to 5-fold by H_2_O. For the temperature gradient experiment in Fig 3, a cDNA from a very high viral load (Ct = 16.0) was further diluted 25-fold by H_2_O and used for the PCR reactions. This dilution and scaling down of the PCR reaction volume, as described below, made the amount of input cDNA approx. 1/300 per reaction compared to the original ARTIC Network’s protocol [7]. For experiments depicted in Figs 1 and 4, three cDNA from clinical samples with moderate viral loads (Ct = 26.5–28.5) was further diluted 2-fold with H_2_O to allow for multiple experiments on the same sample. This dilution and the scaling down of PCR reaction volume made the amount of input cDNA approx. 1/25 per reaction compared to the original ARTIC Network’s protocol. For the multiplex PCR reactions, 1 μl of the diluted cDNA was used in 10 μl reaction mixture of Q5 Hot START DNA Polymerase kit (NEB) (2 μl of 5X buffer, 0.8 μl of 2.5 mM dNTPs, 0.1 μl of polymerase and 0.29 μl of 50 μM primer mix, adjusted by milli-Q water to 10 μl). The thermal program was identical to the original ARTIC protocol: 30 sec polymerase activation at 98°C followed by 30 cycles of 15 sec denaturing at 98°C and 5 min annealing and extension at 65°C (or variable values in gradient mode) in Thermal Cycler Dice ® (Takara Bio). The PCR products in Pool 1 and 2 reactions for same clinical samples were combined and purified with 1X concentration of AMPureXP.

### Sequencing

The purified PCR product was subjected to Illumina library prep using QIAseq FX library kit (Qiagen) in 1/4 scale and using 6 min fragmentation time [16]. After the ligation of barcoded adaptor, libraries were heated to 65°C for 20 min to inactivate the ligase, and then all libraries were pooled in a 1.5 ml tube. The pooled library was first purified by AMPureXP at 0.8X concentration, and then again at 1.2X concentration. The purified library was sequenced for 151 cycles at both paired-ends in Illumina iSeq100.

### Coverage and depth analysis

The obtained reads were mapped to the reference genome of SARS-CoV-2 MN908947.3 [6] using *bwa mem* [17]. The *depth* function in *samtools* [18] with ‘aa’ option was used to determine coverage at each base position. Then, the ‘s’ option of *samtools view* function was used for subsampling reads from each mapping data for normalization.

To calculate coverage (fragment abundance) per PCR amplicon, 98 representative nucleotide (S1 Appendix) each unique to the 98 amplicons amplified by either the V1 or N1 primer set were defined to avoid duplicated count of same amplicon molecules which were fragmented in library preparation. Those representative nucleotides also reside within overlapped regions where original and corresponding alternative primers in the V3 primer set amplify (e.g. the nucleotide for the product 7 overlap with both amplicons amplified by 7_LEFT & 7_RIGHT and 7_LEFT_alt0 & 7_LEFT_alt5). The start and end mapping positions of whole insert sequences of paired-end reads were determined by the *bamtobed* function in bedtools [19] with ‘bedpe’ option. The number of insert fragments overlapping each defined representative nucleotide was counted by the *coverage* function in the bedtools. Fragment inserts of unexpectedly long length (>500 bp from start to end positions) were filtered out from the analysis. The depth counts were summarized and visualized using the python3.6 and the *matplotlib* library [20]. Differential coverage analysis was conducted by edgeR package [21, 22] in R v3 [23] for the three clinical samples as paired-design.

## Supporting information

S1 Table Changes in the N1 primer set from V1 primer set. (XLSX)

S1 Fig Performance comparison of the three multiplex PCR primer sets. Depth plots of the original (V1) and two modified ARTIC primer sets (V3 and N1) for two clinical samples (newly deposited to GISAID with ID EPI_ISL_416749, Ct = 27.3 for A and previously deposited with ID EPI_ISL_416596, Ct = 26.5 for B, each 1/25 input per reaction). Regions covered by amplicons with modified primers and with not modified but interacting primers are highlighted by green and orange colors, respectively. For all data, reads were down-sampled to normalize average coverage to 250X. Horizontal dotted line indicates depth = 30. These two experiments were conducted with the same PCR master mix (except primers) and in the same PCR run in the same thermal cycler.
(PDF)

S1 Appendix BED format file for representative nucleotides used for calculating amplicon abundances. (BED)
