# Supplementary figures and images for: Disentangling primer interactions improves SARS-CoV-2 genome sequencing by multiplex tiling PCR
Source: PLoS One. 2020 Sep 18;15(9):e0239403. doi: 10.1371/journal.pone.0239403 (PMC7500614; doi:10.1371/journal.pone.0239403)

A

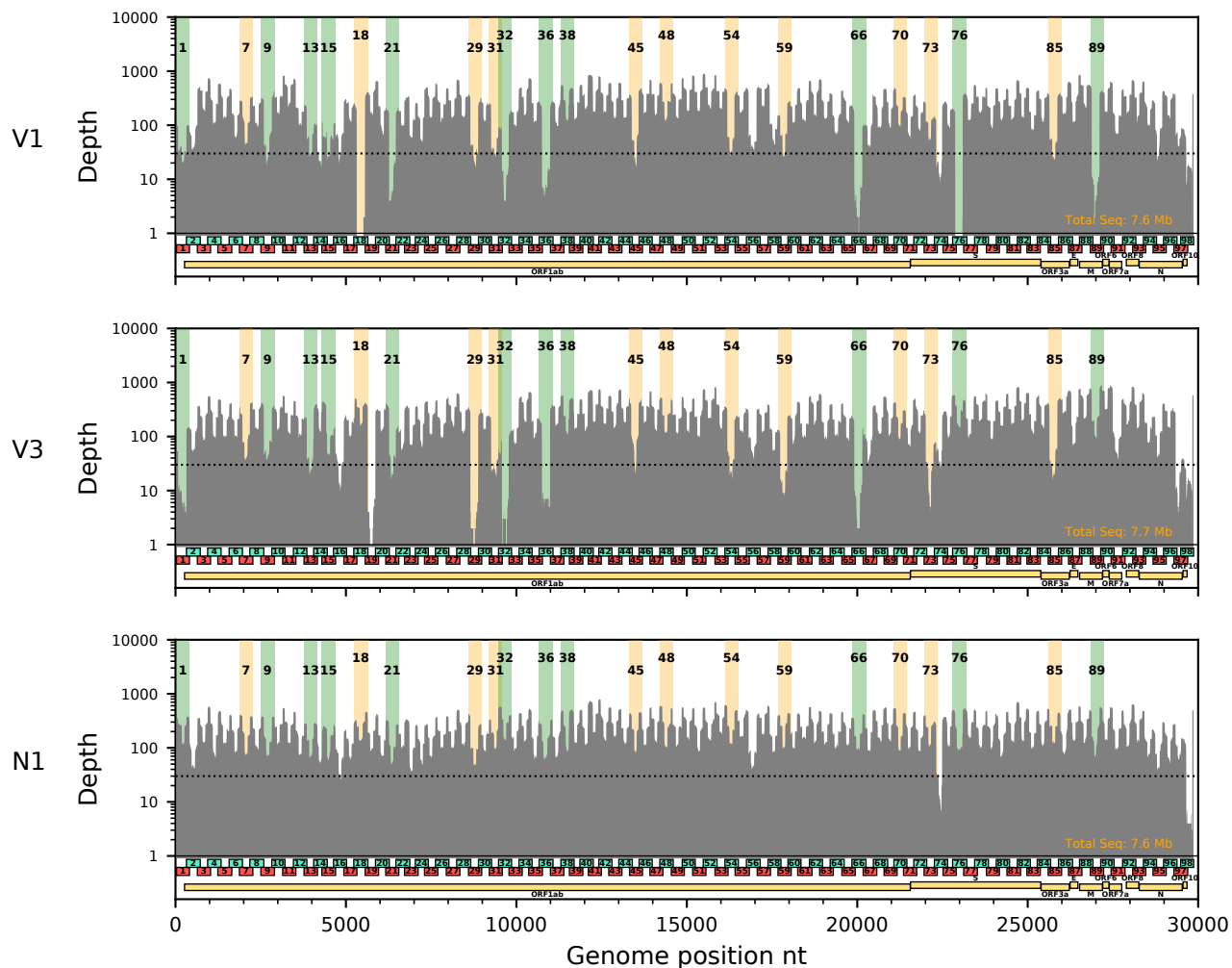

B

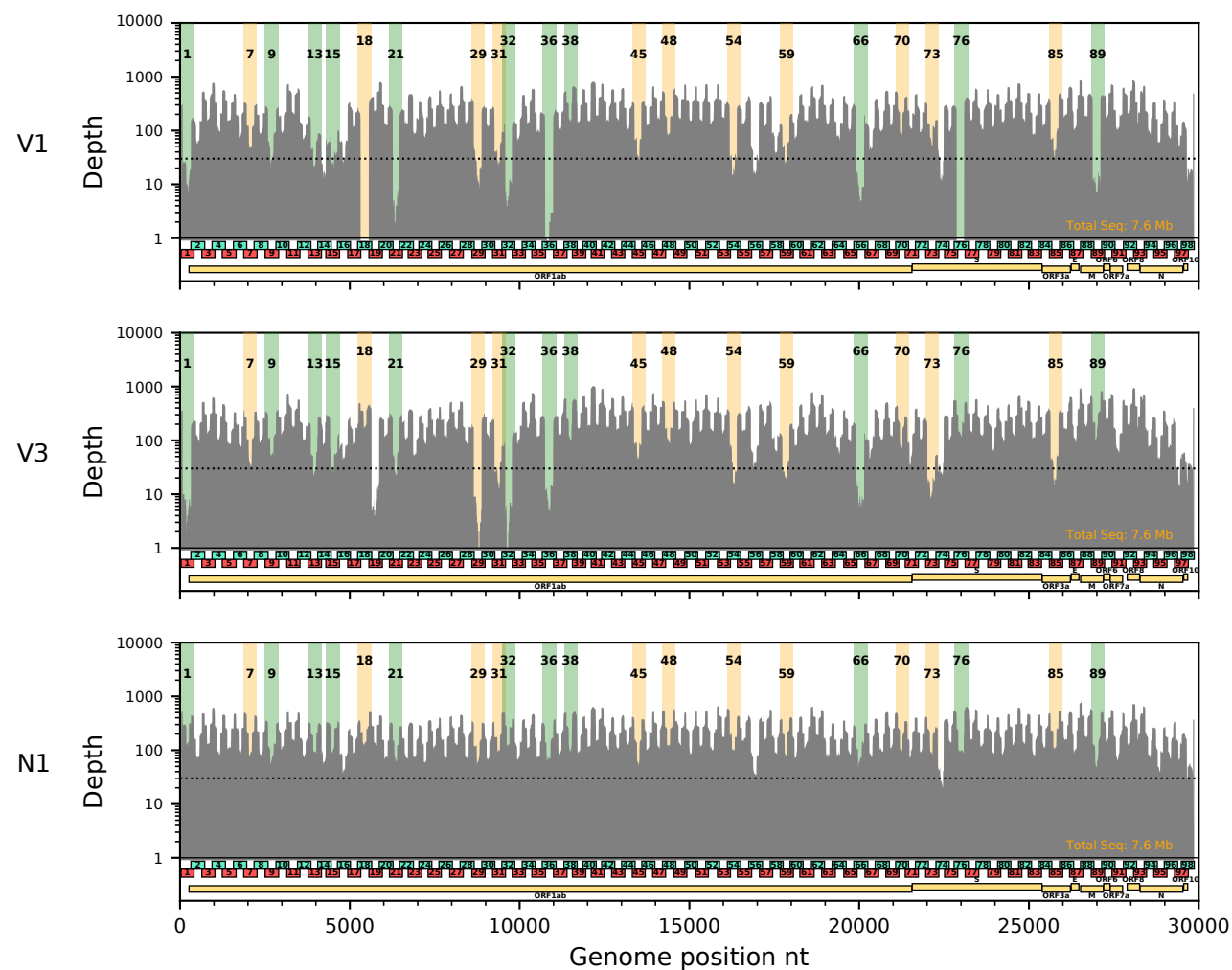

Supplement: S1 Fig — Depth plots of the original (V1) and two modified ARTIC primer sets (V3 and N1) for two clinical samples (newly deposited to GISAID with ID EPI_ISL_416749, Ct = 27.3 for A and previously deposited with ID EPI_ISL_416596, Ct = 26.5 for B, each 1/25 input per reaction). Regions covered by amplicons with modified primers and with not modified but interacting primers are highlighted by green and orange colors, respectively. For all data, reads were down-sampled to normalize average coverage to 250X. Horizontal dotted line indicates depth = 30. These two experiments were conducted with the same PCR master mix (except primers) and in the same PCR run in the same thermal cycler. (PDF) [file pone.0239403.s002.pdf]
